# Supplementary material for: Insights from Real-World Practice: The Dynamics of SARS-CoV-2 Infections and Vaccinations in a Large German Multiple Sclerosis Cohort
Source: Vaccines (Basel). 2024 Mar 3;12(3):265. doi: 10.3390/vaccines12030265 (PMC10976029; doi:10.3390/vaccines12030265)
Supplement: Supplementary file 1 [file vaccines-12-00265-s001.zip › vaccines-2857290-supplementary.pdf]

Supplemental Materials

Insights from real-world practice: Dynamic of SARS-CoV-2 infections and vaccinations in a large German multiple sclerosis cohort

Hernan Inojosa[1a], Dirk Schriefer[1a], Yassin Atta[1], Anja Dillenseger[1], Undine Proschmann[1], Christina Woopen[1], Katharina Schleußner[1], Tjalf Ziemssen[1], Katja Akgün[1*]

Table S1: Demographic and disease-specific description of the patient population by treatment group (n=2115)

	**Age** (years)	**Sex**	**EDSS**	**DMT duration** (years)	**Disease duration**	**Comorbidities**
**Treatment group**	Mean ± SD (median)	n (% female)	Median (IQR)	Mean ± SD (median)	Mean ± SD (median)	n (% with ≥1 comorbidity)
No DMT (n=456)	54.3 ±14.5 (56)	365 (80.0%)	3.5 (2.0–6.0)	-	14.6 ±10.9 (13)	281 (69.7%)
Platform oral (n=267)	44.7 ±11.2 (43)	199 (74.5%)	2.0 (1.5–3.5)	3.9 ±2.9 (4)	9.8 ±8.1 (7)	147 (60.5%)
Platform injectables (n=231)	45.6 ±11.5 (44)	178 (77.1%)	2.0 (1.5–3.0)	7.0 ±5.4 (6)	9.7 ±7.0 (9)	133 (61.3%)
S1PR modulators (n=336)	46.1 ±11.3 (47)	218 (64.9%)	2.5 (1.5–4.0)	4.6 ±3.4 (4)	12.6 ±8.3 (12)	193 (63.1%)
B cell depletion (n=537)	44.4 ±11.9 (44)	358 (66.7%)	3.0 (2.0–5.0)	2.5 ±1.7 (3)	9.9 ±7.8 (8)	296 (60.7%)
VCAM-1 blocker (n=117)	37.3 ±9.1 (35)	95 (81.2%)	2.0 (1.5–3.0)	5.8 ±3.9 (6)	10.9 ±5.9 (11)	42 (37.8%)
Induction therapies (n=114)	42.5 ±10.2 (41)	86 (75.4%)	3.0 (2.0–4.5)	5.5 ±3.3 (5)	12.2 ±6.3 (11)	78 (72.9%)
Others (n=57)	44.4 ±11.5 (47)	34 (59.6%)	3.0 (2.0–4.0)	2.9 ±3.4 (1)	7.2 ±7.4 (5)	34 (65.4%)

Table S2: Number of SARS-CoV-2 vaccinations, infections and infection severity stratified by treatment group and statistically significant differences between groups (n=2115)

		**Number of vaccinations** *(2+ vs 0-1)*		**Number of infections** *(1+ vs 0)*		**Severity of infection** *(non-mild vs mild)*	
	**Treatment group**	n (% with ≥2 vaccinations)	*Pairwise comparisons**	n (% with ≥1 infection)	*Pairwise comparisons**	n (% with non-mild infection)	*Pairwise comparisons**
A	**No DMT**	328 (71.9%)	***E***	130 (28.5%)	***E, F***	25 (27.2%)	-
B	**Platform oral**	208 (77.9%)	-	99 (37.1%)	-	29 (33.0%)	-
C	**Platform injectables**	168 (72.7%)	***E***	76 (32.9%)	-	16 (26.0%)	-
D	**S1PR-modulatiors**	262 (78.0%)	-	124 (36.9%)	-	36 (31.6%)	-
E	**B cell depletion**	447 (83.2%)	***A,C***	227 (42.3%)	***B, A, F***	71 (36.6%)	-
F	**VCAM-1 blocker**	87 (74.4%)	-	37 (31.6%)	***E,A***	8 (29.6%)	-
G	**Induction therapies**	91 (79.8%)	-	32 (28.1%)	***E***	7 (23.3%)	-
H	**Others**	47 (82.5%)	-	25 (43.9%)	-	8 (41.1%)	-

For each treatment group (rows A-H), statistically significant differences ($p<0.05$) to other treatment groups are indicated by the corresponding letters A to H.

*Contrast tests from logistic regression analyses, adjusted for age and sex and using the Bonferroni correction method

Table S3: Logistic regression analysis of severity of infection according to demographic and disease-related factors in people with MS

		**Severity of infection** *[non-mild versus mild]* **univariable**		**multivariable***	
		OR	95%-CI	OR	95%-CI
Age	cont (years)	0.995	0.981-1.010	0.994	0.980-1.009
Age	18-39	Ref		Ref	
	40-59	0.981	0.687-1.400	0.946	0.660-1.354
	60+	0.813	0.455-1.451	0.779	0.435-1.396
Sex	Male	Ref		Ref	
	Female	**0.654**	**0.447-0.958**	**0.647**	**0.441-0.949**
EDSS	cont (points)	1.010	0.912-1.120	1.037	0.917-1.172
EDSS	0-2.5	Ref		Ref	
	3-5.5	1.275	0.877-1.854	1.408	0.939-2.112
	6+	0.977	0.554-1.723	1.116	0.589-2.112
Comorbidity	No	Ref		Ref	
	Single	1.382	0.945-2.021	1.401	0.937-2.096
	Multiple	1.231	0.734-2.064	1.195	0.679-2.103
MS Type	RRMS/CIS	Ref		Ref	
	SPMS/PPMS	0.935	0.569-1.534	0.949	0.543-1.660
MS Type	RRMS	Ref		Ref	
	PPMS	1.080	0.527-2.211	1.023	0.480-2.178
	SPMS	0.864	0.453-1.648	0.905	0.446-1.833
	CIS	2.700	0.716-10.181	2.668	0.701-10.159
Disease duration	cont (years)	1.001	0.979-1.023	1.005	0.981-1.031
Disease duration	0-5	Ref		Ref	
	6-19	1.059	0.723-1.552	1.093	0.738-1.618
	20+	1.123	0.633-1.991	1.285	0.689-2.399
DMT	No DMT	Ref		Ref	
	Platform Oral	1.317	0.695-2.496	1.299	0.675-2.501
	Platform Injectables	0.893	0.431-1.851	0.853	0.405-1.797
	S1P Modulatiors	1.237	0.675-2.267	1.098	0.588-2.049
	B Cell Depletion Therapies	1.547	0.898-2.666	1.486	0.848-2.601
	VL4 Blocker	1.128	0.439-2.904	1.139	0.429-3.025
	Induction Therapies	0.816	0.311-2.136	0.816	0.307-2.174
	Others	1.949	0.703-5.405	1.705	0.603-4.826
Vaccination	0-1	Ref		Ref	
	2+	0.997	0.698-1.426	0.981	0.684-1.405

Bold type: $p<0.05$.
